# Beyond the Basics: Exploring Pharmacokinetic Interactions and Safety in Tyrosine-Kinase Inhibitor Oral Therapy for Solid Tumors

**DOI:** 10.3390/ph18070959

**Published:** 2025-06-26

**Authors:** Laura Veronica Budău, Cristina Pop, Cristina Mogoșan

**Affiliations:** 1 Department of Pharmacology, Physiology and Physiopathology, Faculty of Pharmacy, Iuliu Hatieganu University of Medicine and Pharmacy, 400347 Cluj-Napoca, Romaniacmogosan@umfcluj.ro (C.M.); 2 Amethyst Radiotherapy Center Clinic, 407280 Florești, Romania

**Keywords:** tyrosine kinase inhibitors (TKIs), cancer, drug–drug interactions, safety of TKIs, drug therapy optimization

## Abstract

Cancer remains a major global health burden driven by complex biological mechanisms, and while targeted therapies like tyrosine kinase inhibitors (TKIs) have revolutionized treatment, their efficacy and safety are significantly influenced by drug–drug interactions (DDIs). Tyrosine-kinase receptors (RTKs) regulate critical cellular processes, and their dysregulation through mutations or overexpression drives oncogenesis, with TKIs designed to inhibit these aberrant signaling pathways by targeting RTK phosphorylation. Pharmacokinetic DDIs can critically impact the efficacy and safety of TKIs such as erlotinib, gefitinib, and pazopanib by affecting their absorption, distribution, and metabolism. The modification of pH can influence drug absorption; furthermore, the inhibition or induction of metabolizing enzymes may affect biotransformation, while distribution can be altered through the modulation of transmembrane transporters. Additionally, ensuring quality of life during TKI treatment requires vigilant monitoring and management of adverse events, which range from mild (e.g., rash, diarrhea, fatigue) to severe (e.g., hepatotoxicity, cardiotoxicity). Drug-specific toxicities, such as hyperlipidemia with lorlatinib or visual disturbances with crizotinib, must be assessed using specific criteria, with dose adjustments and supportive care tailored to individual patient responses. Thus, optimal TKI therapy relies on managing drug interactions through multidisciplinary care, monitoring, and patient education to ensure safety and treatment efficacy.

## 1. Introduction

Cancer, a complex group of diseases characterized by uncontrolled cell growth and proliferation, continues to pose a significant global health challenge, with an estimated 19.3 million new cases and nearly 10 million cancer-related deaths reported in 2020 alone, underscoring the urgent need for effective prevention, early detection, and innovative treatment strategies [1]. In 2020, 2.7 million European Union citizens were diagnosed with cancer. Cancer is the second cause of death in the European Union. In 2022, approximately 1.3 million people in the EU died from cancer, according to estimates from the European Cancer Information System (ECIS) and the number of new cancer cases diagnosed annually is estimated to increase by 24% until 2035. In Romania, over 95,000 new cancer cases are diagnosed annually and almost 54,000 deaths occur, with solid tumors entering top five types of cancer for both men and women [2].

Tumor proliferation leading to cancer involves a series of genetic and cellular alterations that disrupt normal regulatory mechanisms. The initial step, known as tumor initiation, typically results from genetic mutations that cause a single cell to proliferate abnormally. This uncontrolled cell division leads to the formation of a clonally derived population of tumor cells. This is often caused by mutations in proto-oncogenes, such as *RAS*, *MYC*, and *EGFR*, which become oncogenes and drive excessive proliferation. In parallel, tumor suppressor genes, like *TP53* and *RB1*, lose their function due to mutations, eliminating critical cell cycle checkpoints and allowing abnormal cells to continue dividing unchecked [3]. As these cells accumulate, they often acquire additional mutations that further enhance their proliferative capacity and survival. A hallmark of cancer cells is their ability to sustain chronic proliferation by altering cell cycle regulation. This is often achieved through the overexpression or hyperactivation of cell cycle-related proteins, which drive continuous cell division [4].

Cancer cells frequently evade apoptosis, the programmed cell death mechanism that eliminates damaged or unneeded cells by upregulating anti-apoptotic proteins such as *BCL-2* and downregulating pro-apoptotic signals. By circumventing apoptosis, these cells gain a survival advantage, allowing them to persist and accumulate further mutations. Sustained angiogenesis also plays a crucial role, as tumors stimulate new blood vessel formation via *VEGF* (vascular endothelial growth factor) to ensure a continuous supply of nutrients and oxygen. Additionally, the tumor microenvironment plays an important role in cancer progression. Interactions between tumor cells and their surrounding stroma, immune cells, and extracellular matrix components can promote tumor growth, invasion, and metastasis [5].

Understanding these mechanisms is essential for developing targeted therapies aimed at inhibiting tumor proliferation and improving cancer treatment outcomes, acting on specific molecular pathways essential for cancer cell survival.

Tyrosine-kinase inhibitors (TKIs) were designed to inhibit the target kinase from catalyzing the transfer of a phosphoryl group from a nucleoside triphosphate donor to the hydroxyl group of tyrosine residues on protein substrates to stop the activation of downstream signaling cascades [6]. Imatinib was the first TKI approved by the Food and Drug Administration (FDA) in 2001 for the treatment of chronic myeloid leukemia (CML) managing to increase life expectancy and transform CML into a chronic disease [7]. Since then, multiple targets have been identified in various tumors, and specific TKIs have been designed to stop phosphorylation and tumor development.

However, TKIs’ effectiveness and safety can be significantly impacted by drug–drug interactions (DDIs). Most of the TKIs are formulated for oral administration and are prone to multiple types of drug interactions. Pharmacokinetic interactions can appear in the absorption phase, due to modifications of gastric pH levels. Acid-reducing agents (proton pump inhibitors, H2 antagonists, antacids) may also impair the solubility and absorption of TKIs like erlotinib and dasatinib, decreasing their bioavailability [8]. Interactions can also appear in the distribution phase, when membrane transporters play an important role and in the metabolization phase, as most of TKIs are metabolized by CYP450 enzymes such as CYP3A4, CYP1A1, CYP1A2, CYP2D6, CYP2C8, and CYP2C9 [9]. Concomitant use of CYP3A4 inhibitors (e.g., ketoconazole, ritonavir) can increase TKI plasma levels, potentially leading to enhanced toxicity, including hepatotoxicity and cardiovascular effects. Conversely, CYP3A4 inducers (e.g., rifampin, phenytoin) can reduce TKI concentrations, diminishing therapeutic efficacy and promoting drug resistance. Additionally, TKIs can act as substrates or inhibitors of drug transporters like P-glycoprotein (P-gp), affecting drug absorption and clearance.

The prevalence of preventable DDIs emphasizes the critical need for healthcare providers to be vigilant, particularly as they manage patients with multiple medications.

Certain risk factors augment the likelihood of experiencing DDIs. Comorbidities, for instance, often necessitate the use of multiple medications to manage different health conditions, increasing the potential of harmful interactions [10]. In this demographic, pharmacokinetic alterations due to changes in metabolism and organ function further exacerbate the risk of DDIs and associated ADRs.

Genetic polymorphisms in drug-metabolizing enzymes and transporters also contribute significantly to an individual’s response to medications, potentially explaining why some patients experience ADRs while others do not, even when prescribed the same drug regimen [11]. This is particularly relevant in the management of chronic diseases where medication regimens are complex and must be closely monitored.

To mitigate the risks associated with DDIs, healthcare professionals are encouraged to assess patient-specific factors such as existing comorbidities, treatment adherence, concurrent medications, and genetic factors influencing drug metabolism. Implementing strategies like therapeutic drug monitoring, careful medication reconciliation, and the use of clinical decision support systems can be instrumental in preventing ADRs stemming from DDIs [12]. Through these means, healthcare providers can enhance therapeutic outcomes while minimizing adverse effects, thereby improving overall patient safety and health management.

In this context, the aim of the present review is to present the main DDIs that can significantly modify the pharmacokinetics and therapeutic outcomes of TKIs, affecting their efficacy and safety profiles, which in turn influence patient survival and quality of life.

## 2. Tyrosine-Kinase Receptors as Therapeutical Targets

Tyrosine-kinase receptors (RTKs) are proteins that play key roles in cell signaling by adding phosphate groups to specific tyrosine residues—a process essential for controlling cell growth, differentiation, and survival. Dysregulations in RTK signals may lead to oncogenesis via multiple mechanisms: overexpression, mutation, or translocation and atypical ligand induction [13]. Some of RTKs’ mutations are called “driver mutations” that promote fast cellular growth of the tumor cells and maintain their survival. EGFRs (epidermal growth factor receptors), VEGFRs (vascular endothelial growth factor receptors), PDGFRs (platelet-derived growth factor receptors), FGFRs (fibroblast growth factor receptors), ROR1 and ROR2 (receptor tyrosine kinase-like orphan receptors 1 and 2), and other RTKs are overexpressed or mutated in solid or hematologic malignancies, activating Ras/MAPK, PI3K/Akt, JAK/STAT, or PLCγ metabolic pathways, thus contributing to enhanced cell proliferation, differentiation, migration, and cell death regulation [14]. TKIs are specifically designed to block the activity of these receptors. They usually target intracellular ATP-binding sites of RTK, leading to the inhibition of phosphorylation and blocking of the signaling cascade [15]. Tyrosine kinase inhibitors (TKIs) have emerged as pivotal therapeutic agents in the management of various solid tumors, significantly altering the treatment landscape for malignancies, including non-small-cell lung cancer (NSCLC), gastrointestinal stromal tumors (GISTs), and other sarcomas. In NSCLC, drugs such as gefitinib and erlotinib target the epidermal growth factor receptor (EGFR) pathway, which is frequently altered in solid tumors, leading to enhanced tumor sensitivity and reduced aggressiveness [16]. Moreover, TKIs like entrectinib have been approved for treating tumors with neurotrophic receptor tyrosine kinase (NTRK) fusions, highlighting the promise of targeted therapy based on specific genetic alterations [17].

A summary of TKIs approved by the EMA for the treatment of various solid tumors, with provided molecular targets and indications, is provided in Table 1.

## 3. Pharmacokinetic Drug–Drug Interactions

**Figure 1:**
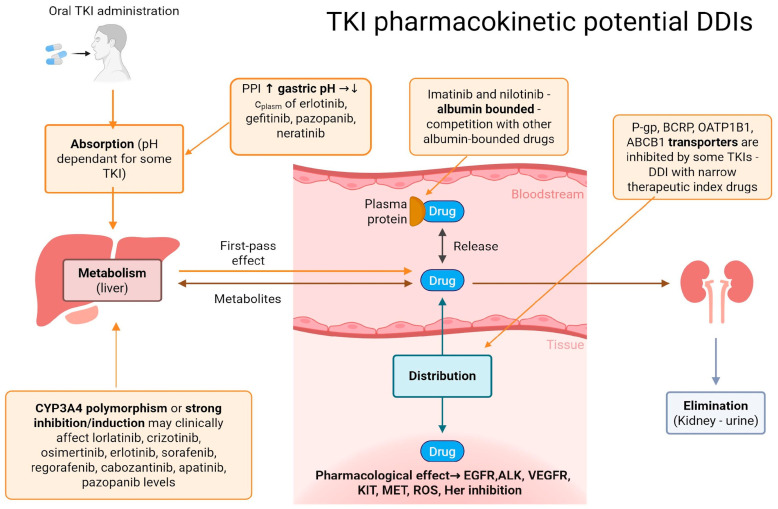
TKI potential pharmacokinetic DDIs. https://BioRender.com accessed on 11 June 2025. PPI—Proton Pump Inhibitor, P-GP—P-glycoprotein, BCRP—Breast Cancer Resistance Protein, OATP1B1—Organic Anion Transporting Polypeptide 1B1, ABCB1—ATP Binding Cassette Subfamily B Member 1, TKI—Tyrosine Kinase Inhibitor, DDI—Drug–Drug Interaction, EGFR—Epidermal Growth Factor Receptor, ALK—Anaplastic Lymphoma Kinase, VEGFR—Vascular Endothelial Growth Factor Receptor, KIT—Stem Cell Factor Receptor, MET—Hepatocyte Growth Factor Receptor, ROS—c-ros Oncogene 1, HER—Human Epidermal Growth Factor Receptor.

Pharmacokinetic DDIs involving TKIs present significant challenges in clinical oncology. TKIs are often associated with considerable variability in their pharmacokinetic properties due to multiple factors including metabolism by cytochrome P450 liver enzymes and interactions with transport proteins. Such interactions may lead to altered drug levels in patients, resulting in either subtherapeutic effects or increased toxicity, effectively complicating treatment regimens and patient outcomes. Figure 1 summarizes the potential TKI drug–drug interactions (DDIs).

### 3.1. DDIs Affecting TKI Absorption

TKIs are weak bases that need a low pH for good solubility and absorption. Proton pump inhibitors (PPIs) are commonly prescribed to manage gastric acid-related conditions in cancer patients, but their ability to elevate intragastric pH may impair the solubility and absorption of various TKIs, including erlotinib and gefitinib, which are particularly sensitive to pH changes. Studies have shown that the co-administration of PPIs can reduce the plasma concentration of these TKIs, leading to diminished therapeutic effects [18]. This may lead clinicians to increase TKI doses to compensate, potentially resulting in dose-dependent liver toxicity. Additionally, both PPIs and TKIs are metabolized by cytochrome P450 enzymes, particularly CYP3A4 and CYP2C19. PPIs can inhibit or induce these enzymes, altering TKI metabolism and potentially leading to the accumulation of the parent drug or toxic metabolites, which may cause hepatocellular injury through oxidative stress or mitochondrial dysfunction. Furthermore, PPIs may inhibit hepatic transporters like BCRP, P-glycoprotein, and MRP2, impairing biliary excretion and contributing to cholestatic liver injury. In some cases, idiosyncratic hepatotoxicity—possibly immune-mediated or due to reactive metabolite formation—can be exacerbated by altered drug exposure, resulting from these interactions. Clinically, hepatotoxicity may manifest as elevated liver enzymes or bilirubin levels, typically emerging within weeks of initiating therapy, and often resolves after discontinuation of the TKI or PPI (Table 2). As a safety measure, PPI use is recommended when needed, for the shortest period possible [19]. For example, the administration of lansoprazole with neratinib decreased neratinib exposure in healthy subjects due to decreased solubility at an increased gastric pH and long-acting proton pump inhibition by lansoprazole [20]. Concomitant use of PPI and pazopanib has a significant negative impact on oncologic survival endpoints (OS—overall survival and PFS—progression-free survival), thus decreasing the efficacy of TKI treatment [21].

Potential absorption DDIs are not always corelated with clinical effects. In the case of osimertinib, the administration of omeprazole did not affect exposure to metabolites, compared to osimertinib administered alone, probably because of the pharmacokinetic particularity of osimertinib, which exhibits quick dissolution and higher solubility at pH 1 to 6.8 compared to pH > 7 [22]. Although cabozantinib is a weak base that is potentially affected by gastric pH modifications, a retrospective review showed no differences in efficacy and safety for patients with metastatic renal cell carcinoma (mRCC) who used PPIs during cabozantinib treatment compared to those who did not [23].

### 3.2. DDIs Affecting TKI Distribution

TKIs, such as imatinib and nilotinib, are primarily bound to plasma proteins, particularly albumin, which plays a crucial role in their distribution throughout the body. The presence of other drugs that also bind to these proteins in a high percentage can lead to competitive inhibition, resulting in altered free concentrations of TKIs and their pharmacological effects.

Moreover, variations in the expression of transporters, such as those from the ATP-binding cassette (ABC) family, can also affect the tissue distribution of TKIs, leading to suboptimal therapeutic effects. For example, changes in the activity of transporter proteins like ABCG2 can limit the ability of TKIs to reach target tissues effectively [24]. Glycoprotein P (P-gp) and breast cancer resistance protein (BCRP) are the main ABC transporters involved in TKI resistance and inter-individual variation [25]. The association of TKI and P-gp or BCRP inhibitors may be a solution to overcome tumor resistance to these drugs and to increase TKI availability including at the central nervous system (CNS) level. Some of the TKIs like imatinib, dasatinib, nilotinib, erlotinib, gefitinib, and lapatinib are both substrates and inhibitors of P-gp and BCRP and can be used to sensitize resistant cells against conventional cytotoxic agents in BCRP-transduced cells rather than parental malignant cells. Understanding these distribution phase interactions is crucial for optimizing TKI therapy in cancer management, as this knowledge enables oncologists to adjust treatment regimens and monitor for potential complications arising from concurrent medications [26]

Wind et al. investigated the influence of rifampicin, a potent P-gp inducer, and ritonavir, a strong P-gp inhibitor, on afatinib, an EGFR-TKI and a known P-gp substrate. Their findings indicated a significant increase in the maximum plasma concentration (c_max_) of afatinib following ritonavir administration; however, the overall plasma concentration–time profiles remained comparable between afatinib alone and its co-administration with ritonavir. Conversely, rifampicin reduced afatinib c_max_, yet no significant alterations were observed in the plasma concentration curve. Given that afatinib does not exhibit substantial susceptibility to efflux transporter interactions or CYP450-mediated metabolism, its potential for DDIs is considered minimal, supporting its use in combination therapy [27].

Osimertinib was evaluated as a potential P-gp inhibitor because of its ability to activate pregnane X receptor (PXR), which regulates P-gp expression. Using fexofenadine as a substrate, osimertinib proved to be a weak P-gp inhibitor, concomitant administration with P-gp substrates being generally safe, with the mention that drugs with a narrow therapeutic index like digoxin, aliskiren, or dabigatran should be associated with caution [28].

Concerning interactions with other transporters, such as organic anion transporting polypeptide 1B1 (OATP1B1), pazopanib is an example of a strong inhibitor, but its effect is short-lasting and is preincubation-dependent, showing a low potential of OATP1B1-mediated DDI in clinical practice [29]. In vitro studies have shown the inhibitory potential of crizotinib upon ABCB1 transporter efflux, which may be used clinically in enhancing the cytotoxicity of agents transported by ABCB1 and reversing drug resistance mediated by ABCB1 transporters [30]. Tivozanib and axitinib are BCRP inhibitors, and dose adjustments are required for BCRP substates. Tivozanib is a third-generation VEGF TKI, which has better patient adherence and good efficacy due to its low DDI potential, administration regardless of food intake, and once/day administration [31]. Association of TKIs and DOACs is common in cancer patients, to prevent venous thromboembolism, but TKIs’ influence upon P-gp and CYP3A4 affects DOAC (dabigatran, apixaban, rivaroxaban, edoxaban) plasmatic concentrations. Imatinib and crizotinib significantly increase the AUCs of all mentioned DOACs; tucatinib has moderate effects, while nilotinib, lapatinib, ceritinib, cabozantinib, osimertinib, and sunitinib present a mild potential for interactions. However, the association presents a high risk of bleeding, which justifies the preference for certain compounds with a lower potential for drug interactions, such as apixaban or edoxaban; and in cases where such interactions cannot be avoided, clinicians should adjust the dose, perform careful monitoring, or modify the treatment [32].

### 3.3. DDIs Affecting TKI Metabolization

Before any DDIs, the first-pass metabolism of TKIs also plays a significant role in their pharmacokinetics and can substantially influence metabolization and bioavailability, even in the context of an acceptable absorption of these drugs in patients. Upon oral administration, TKIs undergo extensive metabolism, primarily in the liver, predominantly mediated by cytochrome P450 enzymes, particularly CYP3A4. This biotransformation leads to the formation of both active and inactive metabolites, which can potentially affect therapeutic efficacy and toxicity profiles. As a consequence, the concentration of the parent drug in the systemic circulation can be significantly reduced, influencing the plasma levels attainable in patients [33]. In oncology patients, who frequently exhibit an altered hepatic functionality, this first-pass effect can further complicate TKI dosing. Variability in hepatic transporter proteins, such as OATPs (organic anion transporting polypeptides), may modulate drug absorption and elimination, resulting in altered pharmacokinetic profiles that require careful dose adjustments based on individual metabolic status [34]. Furthermore, certain acid-reducing agents (ARAs) can influence the solubility and, consequently, the absorption of TKIs due to their pH-dependent solubility, potentially exacerbating variability in bioavailability [35]. This interplay between first-pass metabolism and systemic distribution underscores the necessity of individualized therapeutic strategies in TKI administration to optimize clinical outcomes while minimizing adverse effects [34].

Additionally, interactions with other medications that induce or inhibit these enzymes can lead to significant alterations in TKI plasma concentrations. Understanding these interactions is vital for oncologists, as avoiding or managing these DDIs through the careful selection of concomitant therapies and monitoring strategies can enhance patient outcomes, minimize adverse effects, and maintain effective cancer control. Cytochrome polymorphism may lead to major interactions in poor metabolizers and to intra-individual variation, which should be assessed before starting the treatment to ensure safety and efficacy. In some cases, genetic variability in metabolizing enzymes can increase toxicity, which in turn can be augmented by different types of DDIs. Genotyping is an important step in precision medicine and individualized treatment and should be the standard of care [36]. The effect of CYP3A4/5 and ABC transporter genetic polymorphism on lenvatinib was studied in an Asian population, both on healthy and thyroid-cancer patients. Li et al. administered lenvatinib to a cohort of 32 healthy Chinese patients and determined the CYP3A4/5 (CYP3A4*1G, CYP3A5*3) and ABC transporter (ABCB1 (3435 C>T, 1236 C>T, 2677 G>T/A), ABCG2 (421 C>A, 34 G>A), and ABCC2-24 C>T) genotypes. The only statistically relevant polymorphism was ABCB1 3435 C>T, a gene that codifies P-gp, with its mutation leading to an increase in AUC with higher toxicity risks [37]. Ozeki et al. determined the influence of CYP3A4/5 and ABC transporter polymorphisms on lenvatinib in Japanese patients with thyroid cancer. Patients with both the CYP3A4*1/*1 and ABCC2 −24T alleles exhibited 1.5-fold higher lenvatinib values than those in patients with both the CYP3A4*1G/*1G and ABCC2 −24C/C genotypes, which may be associated with a higher incidence of adverse reactions. Details of the CYP3A4 genotype would be helpful to assess the initial dose of lenvatinib, which ensures optimal plasmatic concentrations [38]. The CYP2D6 polymorphism (CYP2D6 allele *5 or *10) can impact NSCLC treatment with gefitinib, as Takimoto et al. determined in a cohort of 55 Japanese patients. As severe hepatotoxicity was observed in 25% of Japanese patients taking gefitinib, genotyping CYP2D6 was performed to assess if there is a direct impact. No direct correlation was found, but to a certain extent, there is a contribution of the reduced CYP2D6 function to hepatotoxicity [39]. Another isoenzyme that is susceptible to pharmacogenomic variations is CYP2C9, whose poor metabolizers (CYP2C9 *3/*3 genotype) present higher plasmatic concentrations and a higher risk of adverse reactions [40].

#### 3.3.1. DDIs Affecting ALK TKIs

Loratinib, a third-generation ALK/ROS 1 TKI, is metabolized by CYP3A4 and thus prone to DDIs. Patel et al. evaluated itraconazole’s effect, a strong CYP3A4 inhibitor, upon lorlatinib in healthy patients: multiple daily doses of itraconazole significantly increased lorlatinib levels. It is recommended to avoid co-administration with strong CYP3A4 inhibitors, or if that is not possible, to lower the lorlatinib dose from 100 mg to 75 mg [41]. Crizotinib, an ALK (anaplastic lymphoma kinase) and c-met TKI, is metabolized by CYP3A4 and is also a P-gp substrate. Co-administration of rifampicin, a potent CYP3A4 inducer, significantly decreased bioavailability, while co-administration of ketoconazole, a potent CYP3A4 inhibitor, increased crizotinib exposure by 3.4-fold [42]. Hurtado et al. evaluated the effect of ceritinib on CYP3A4 and CYP2C9 substrates. The ALK inhibitor is a strong CYP3A4 inhibitor, increasing midazolam levels, and a weak CYP2C9 inhibitor. Still, warfarin co-administration, as a narrow-therapeutic-index drug, should be carried out with dose reduction and increased INR monitorization [43]. The potential of alectinib to induce or inhibit was evaluated in vitro and it has a low impact upon CYP3A4, CYP2B6, and CYP1A2 isoenzymes, with the potential to cause DDIs [44]. Morcos et al. also checked for alectinib’s clinically relevant DDIs through CYP3A4 induction or inhibition. Co-administration of posaconazole, a strong CYP3A4 inhibitor, had a minor effect on plasma concentrations, while rifampicin, a strong CYP 3A4 inducer, also had a minor effect on the plasma levels of both alectinib and its active metabolite [45]. Nakagawa et al. also concluded that alectinib has low DDI risk potential, being a highly lipophilic compound, with low urinary excretion, metabolized by many enzymes, not only CYP3A4, which has only 40–50% contribution [46].

#### 3.3.2. DDIs Affecting EGFR TKIs

Osimertinib, a third-generation EGFR-TKI, is metabolized by CYP3A4/5, and co-administration with strong inducer/inhibitors may modify its bioavailability. Vishwanathan et al. dosed serum osimertinib levels after the administration of a strong inducer, rifampicin, and a strong inhibitor, itraconazole. Administration of osimertinib with itraconazole did not clinically affect osimertinib levels, but rifampicin decreased osimertinib levels with clinical relevance. Co-administration of osimertinib with strong CYP3A4 inhibitors is safe, while strong inducers should be avoided. If it is not possible, a dose escalation to 160 mg instead of 80 mg is recommended [47]. Gefitinib is a strong inducer of CYP1A1, an enzyme highly expressed in pulmonary tumor tissues. When associated with ningetinib, another TKI used in NSCLC, it did not affect the pharmacokinetics of the latter, although it is metabolized by CYP1A1, but more safety studies should be performed on NSCLC patients [48]. Erlotinib is an EGFR-TKI used for the treatment of NSCLC and pancreatic cancer. It is metabolized by CYP3A4 and interacts with strong CYP3A4 inhibitors/inducers, P-gp inhibitors, coumarin-derived anticoagulants, and cigarette smoke (for smokers—approx. 30 cigarettes/day, initial dose should be 300 mg instead of 150 mg for non-smokers) [49].

#### 3.3.3. DDIs Affecting VEGF TKIs

VEGF TKIs used in hepatocellular carcinoma (HCC) treatment, lenvatinib, sorafenib, regorafenib, and cabozantinib are metabolized by CYP3A4, but not all strong inducers/inhibitors have a clinical impact upon them. Strong CYP3A4 inducers should be avoided during sorafenib, regorafenib, and cabozantinib treatment, but can be used during lenvatinib treatment, while strong CYP3A4 inhibitors may be used during treatment with sorafenib and lenvatinib, but should be avoided when regorafenib and cabozantinib are administered [50]. Sorafenib and lenvatinib are only partially metabolized by CYP3A4 and also rely on alternative routes such as glucuronidation (UGT1A9) and excretion, making them less sensitive to CYP3A4 inhibition. As a result, co-administration with strong CYP3A4 inhibitors may cause only moderate increases in drug levels, allowing for cautious use with possible dose adjustments. In contrast, regorafenib and cabozantinib are primarily metabolized by CYP3A4, and strong inhibitors can significantly raise their plasma concentrations, increasing the risk of serious toxicities such as hepatotoxicity, hypertension, and hand–foot syndrome. Cabozantinib is a CYP2C9 and CYP2C19 inhibitor, and its association with warfarin led to bleeding and increased INR for a 64-year-old patient with mRCC. Vitamin K doses and temporary discontinuation of the mRCC treatment normalized the INR, but the patient decided to stop nRCC treatment and was referred to hospice care [51].

VEGF TKIs used in renal cell carcinoma (RCC) treatment, sunitinib, sorafenib, lenvatinib, cabozantinib, pazopanib, axitinib, and tivozanib, are also metabolized by CYP3A4, but not all interactions are clinically relevant: lenvatinib is not affected by strong CYP3A4 inhibitors or inducers, sorafenib and tivozanib are not affected by CYP3A4 inhibitors, while the association with inducers of all drugs except lenvatinib should be avoided or dose adjustments should be made [31]. Apatinib metabolism is catalyzed by CYP3A4 and CYP3A5, and co-administration with strong inducers (rifampicin) increased its pharmacokinetics by 5-fold, while strong inhibitors (itraconazole) had a weak effect (only 1.5–2-fold decrease) [41].

#### 3.3.4. DDIs Affecting Multiple Target TKIs

Plasmatic levels of imatinib may vary due to CYP enzyme polymorphism, demographic factors, low compliance, or DDIs, and measurements may be useful when suspecting toxicities, non-compliance, DDIs, or a suboptimal therapeutic response [52]. Pazopanib is metabolized by CYP3A4 mainly, followed by CYP1A2 and CYP2C8, but it is also a substrate for P-glycoprotein and BRCP, contributing to elimination into the gastrointestinal tract through biliary secretion [53]. Concomitant administration with clarithromycin, an antibiotic prescribed for acute bronchitis and a strong CYP3A4 inhibitor, led to hepatic failure and death in a 44-year-old renal cancer patient [54]. Brigatinib is a TKI that has multiple targets: ALK, ROS 1, and EGFR resistance mutations. It is metabolized by CYP3A4 and CYP2C8 and inhibits CYP3A4. It is indicated for patients with NSCLC who have ALK mutation (ALK+ NSCLC), developed progression under crizotinib, and have increased CNS penetration properties, targeting brain metastases. High plasma levels may increase brigatinib adverse reactions such as nausea, fatigue, diarrhea, creatine–phosphokinase (CPK) elevations, headaches, dyspnea, hypertension, and pulmonary-related events, in specific situations, as reported, leading to treatment interruptions or discontinuation [55]. In vitro and in vivo studies on rats have shown that exposure to rivaroxaban increases when they are administered imatinib because of CYP2J2, CYP3A4, BCRP, and P-gp inhibition and decreases when they are administered sunitinib due to BCRP induction, which may impact cancer-associated venous thromboembolism management [56].

Another TKI, neratinib, is an irreversible Her1, Her2, and Her4 inhibitor used in the treatment of Her2+ breast cancer. Its solubility is pH-dependent, with concomitant administration of PPIs lowering its bioavailability by 60–70%. Its metabolization is CYP3A4-dependent, and co-administration of strong inhibitors or inducers should be avoided. Patients that are under treatment with drugs with a narrow therapeutic index and that are transported by P-gp (like digoxin, cyclosporine, methotrexate, and acenocoumarol) need to be monitored because of neratinib P-gp inhibition [57].

During the COVID-19 pandemic, a lot of treatment options were used to control the symptoms and lower mortality and morbidity. Antiviral drugs (remdesivir, lopinavir/ritonavir combination, umifenovir, and favipiravir) were the first choice, followed by antibiotics, antimalarial drugs (hydroxychloroquine, chloroquine), antiparasitic drugs (ivermectin), corticosteroids, interferon, and IL-6 monoclonal antibodies (tocilizumab) [58,59]. When initiating treatment for a new acute disease like COVID-19, the risk of DDIs should be assessed in patients receiving TKIs or supportive treatment for nausea, vomiting, GI or dermatologic toxicities, and/or pain [60].

Table 3 summarizes the DDIs identified during TKI metabolization and offers insights into proper DDI management, according to cited articles and drugs’ SPC (Summary of Product Characteristics).

#### 3.3.5. Potential Interactions with Complementary and Alternative Medicines

Cancer patients often use complementary and alternative medicine (CAM) treatments to alleviate symptoms, to improve immunity, to feel empowered in their treatment decision, or just because they so not want to miss any chance in their fight with cancer. It is estimated that over 50% of cancer patients are using CAMs, most of them female, with high education, a high income, or previous CAM use [61]. Although the evidence is not as strong as for drugs, probably because of the legislative gap and the differences in how producers can register a supplement in various countries, CAMs may interact with cancer treatment. A TKI used in melanoma may interact with CAM, vitamins, selenium, and other supplements with active principles more than homeopathics, hormones, mushrooms, Chinese teas, and mistletoe [62].

Rodseeda et al. determined the inhibitory effects of *Andrographis paniculata*, *Curcuma zedoaria*, *Ganoderma lucidum*, *Murdannia loriformis*, and *Ventilago denticulata* extracts on the metabolism of gefitinib, lapatinib, and sorafenib. *Curcuma zedoaria* extract potently inhibited CYP3A-mediated lapatinib and sorafenib metabolism, respectively, while the metabolism of gefitinib was strongly inhibited by *Murdannia loriformis* and *Ventilago denticulata* extracts. *Andrographis paniculata* and *Ganoderma lucidum* hydroalcoholic extracts had less effect on the metabolism of the tested anticancer drugs [63].

St. John’s Worth (*Hypericum perforatum*) is a well-known CYP3A4 and CYP2C9 inducer affecting several TKIs: erlotinib, dabrafenib, gefitinib, imatinib, nilotinib, pazopanib, sorafenib, and sunitinib; it decreases plasmatic concentrations by increasing the clearance and accelerating metabolism, which may lead to treatment failure. Panax ginseng inhibits CYP3A4 and CYP2C9, and several case reports mention an increase in imatinib hepatic toxicity, recommending the avoidance of concomitant use [64].

Grapefruit (*Citrus maxima*), bitter orange (*Citrus x auriantum*), turmeric (*Curcuma longa*), aloe vera (*Aloe vera*), ginseng (*Panax ginseng*), and ginger (*Zingiber oficinale*) may increase toxicity due to CYP3A4 inhibition or affect the efficacy because of the lower absorption of some oral anticancer drugs. Using herbal–drug interaction databases such as Hédrine^®^ or the MSKCC database^®^ allows pharmacists to assess potential interactions and leads to pharmaceutical interventions such as herb discontinuation, therapeutic monitoring, pharmaceutical advice, or drug intake optimization [65].

In addition to interactions that may alter pharmacokinetics with undesirable effects, the presence of beneficial interactions with natural products cannot be denied. Resveratrol, found in grapes, has shown capabilities to potentiate the effects of TKIs by modulating apoptosis pathways and reducing cellular resistance to anticancer therapies [66]. Research indicates that resveratrol can enhance the sensitivity of cancer cells to the effects of EGFR-targeted therapies by influencing tumor microenvironment factors and cytokine signaling pathways [67]. Quercetin, another dietary polyphenol prevalent in many fruits and vegetables, exhibits both antioxidant and anti-inflammatory properties, which may improve the therapeutic efficacy of TKIs by reducing drug resistance mechanisms in tumors [68].

Collectively, these compounds serve not only as adjuncts to enhance TKI action but also to mitigate potential side effects, thereby improving overall patient outcomes in cancer therapy. Their multifaceted roles in cancer biology, coupled with their generally favorable safety profiles, make them intriguing candidates for further investigation in combination therapy protocols with TKIs.

Patients should be encouraged to discuss CAM with their oncologists. In turn, oncologists should acknowledge patients’ perspectives and provide informed guidance to ensure the safe integration of CAM. Furthermore, oncology professionals should receive dedicated training in both the evidence supporting CAM and effective communication strategies regarding its use [69].

## 4. TKI Safety

Ensuring quality of life is very important during oncological treatments, and attention to incidences and appropriate adverse event management is crucial. About 47.4% of patients on TKIs encountered at least one potential TKI–drug interaction, with 30.8% of these classified as major interactions that could lead to significant clinical consequences [70]. The adverse events are grouped into five categories, depending on their severity, following the Common Terminology Criteria for Adverse Events (CTCAE), a tool developed by the National Cancer Institute for a harmonized interpretation of toxicity assessment by clinicians [71]. This system categorizes adverse events into five grades of severity, such as grade 1 (mild) including asymptomatic or mild symptoms, based on clinical or diagnostic observations only, and where no intervention is required. Grade 2 (moderate) AEs are minimal and local, noninvasive intervention is indicated. Grade 3 (severe) AEs are medically significant but not immediately life-threatening, which lead to hospitalization or a prolongation of hospitalization and can be disabling. Grade 4 (life-threatening) AEs need urgent intervention and represent an immediate risk to life or to organ function. Finally, grade 5 AEs are death-related [71].

The CTCAE v5.0 system facilitates comparability between clinical trials and real-world data by providing a clear framework for toxicity assessment. Its use ensures that clinicians and researchers can communicate findings reliably and apply consistent thresholds for dose adjustments, treatment discontinuation, or supportive care measures [71].

**Figure 2:**
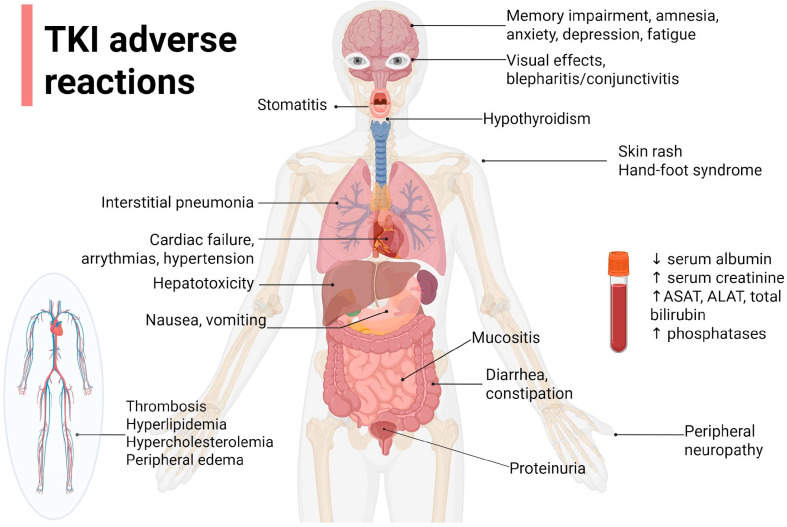
The most common adverse reactions associated with tyrosine kinase inhibitors (TKIs). Created in https://BioRender.com, accessed on 11 June 2025.

Generally, TKIs’ safety profiles are acceptable, with drug- or class-specific toxicities that should be discussed with the patient at initiation and during the treatment. Generally, grade 2 or greater adverse events (AEs) lead to dose interruption or dose reduction, and depending on the severity of the AE or the impairment developed, treatment may continue at a lower dose when the AE is evaluated under grade 2. The assessment is carried out by the clinician because even if pain, for example, is evaluated by the patient at a higher scale, the impact may be considered mild, and symptomatic treatment may be undertaken concomitantly with the target treatment to reach the therapeutic goal [72]. Figure 2 highlights the most common TKI AEs.

### 4.1. Safety of ALK TKIs

Crizotinib is generally well tolerated by ALK + NSCLC patients. Its common AEs are visual effects (flashing lights on moving objects when changing from dark to light), nausea, diarrhea, constipation, vomiting, and peripheral edema, while AEs that usually led to a dose reduction included an increased ALAT, QT prolongation, and neutropenia [73]. When initializing the treatment, clinicians should check serum Ca^2+^, Mg^2+^, and K^+^ levels and liver function (ASAT, ALAT, bilirubin) and take a complete blood count and an EKG, while long-term surveillance includes testosterone dosing (for the evaluation of hypogonadism) and an albuminemia assay (for edema evaluation). Patients should be counselled about the dietary changes needed if diarrhea or constipation occurs, should be warned about the visual disturbances and edema appearance (compression socks are preferred to diuretics, because of electrolyte perturbances) that may impair driving or work, and should be encouraged to communicate any pulmonary or cardiac abnormalities [74]. Lorlatinib, a third-generation ALK-TKI, developed to conquer acquired tumor resistance of generations 1 and 2 of ALK TKIs, was designed to penetrate blood–brain barriers with high potency on cancer cells that mutated [75]. Its common AEs include hyperlipidemias (hypercholesterolemia, hypertriglyceridemia); changes in cognitive function (memory impairment, amnesia), mood (anxiety, irritability, depression), speech (difficulty in finding words, slowed speech), and body weight (increase up to 10–20%); edema (in 57% of the patients), and peripheral neuropathy (onset 6–8 weeks). Lipid levels should be assessed at the baseline, and periodical monitoring is needed; 80% of patients needed at least one lipid reduction drug (rosuvastatin being the drug of choice because of its low CYP interaction potential). Most AEs are mild and reversible if managed well by the multidisciplinary healthcare team [76].

### 4.2. Safety of EGFR TKIs

The EGFR TKI safety profile is balanced, with the specific main adverse reactions being nausea, diarrhea, rashes, abdominal distension [77], and skin toxicity, probably due to the inhibition of intracellular kinase signaling pathways leading to a proliferation of keratinocytes and proinflammatory cytokines, changes in T lymphocyte activity, or secondary infections [78]. Diarrhea is managed by dietary changes and loperamide, and in severe cases, atropine or octreotide; a rash is managed with topical creams with or without corticosteroids, or in severe cases, doxycycline and isotretinoin; stomatitis/mucositis requires good oral hygiene with alcohol-free mouthwash, oral lidocaine, or even antalgic treatment; while paronychia is treated with topical clobetasol or antibiotics/antiseptic combinations [79]. The AURA 1 and 2 studies show that osimertinib, a third-generation EGFR-TKI (including EGFR T790M resistance mutation), is well tolerated, with diarrhea, rash, nausea, and a decrease in appetite as common adverse reactions. In a previous study, 13–21% of patients experienced grade-3 or higher toxicities and 2% presented interstitial lung disease [80]. Patients with chronic hepatitis B that undergo osimertinib treatment should be checked regularly by testing HBV (hepatitis B virus) markers to prevent virus reactivation. A case report of a 69-year-old patient with inactive HBV presented a hepatitis flare 8 months after initializing treatment with osimertinib. An association of lamivudine was added and the ALT of the patient normalized [81]. Another case was of a 68-year-old patient with NSCLC, treated for 4.5 months with osimertinib, who experienced grade-4 hepatotoxicity with liver decompensation and with hyperbilirubinemia, hypoalbuminemia, coagulopathy, and hepatic encephalopathy, which were followed by death after 7 days because of an unidentified HBV reactivation [82].

### 4.3. Safety of MET-TKIs

MET-TKIs (tepotinib, capmatinib, savolitinib, and crizotinib) are usually well tolerated, and common AEs are fluid retention (peripheral edema, hypoalbuminemia, pleural effusion), gastrointestinal toxicities (nausea, vomiting, diarrhea, constipation), and increased serum creatinine and hepatic transaminases and phosphatases. A particularity of MET-TKIs is that they inhibit creatinine transporters by 20%, meaning that increased serum creatinine does not necessarily reflect renal impairment. If the variations are quickly reversible (rapid onset when initiating treatment and rapid decrease when stopped), the creatinine increase is most probably due to transporter inhibition, rather than renal impairment [83].

### 4.4. Safety of Multiple Target TKIs

Cabozantinib is used in mRCC alone or in combination with nivolumab, with tropism for bone metastasis. Its safety profile is similar to other TKIs, the most common side effects being fatigue, diarrhea, hypertension, hand–foot syndrome, coagulation disorders, and proteinuria. In the long term, weight should be monitored, with grade-2 weight loss being reported in 44% of patients using cabozantinib [84]. The usual AEs of sorafenib administration include diarrhea, arterial hypertension, cutaneous toxicity (hand–foot skin reaction and rash), and fatigue. Hypertension should be managed with caution given to DDIs and sorafenib pharmacokinetics: calcium channel blockers like amlodipine or felodipine are safe, while diuretics should be avoided because of the renal failure risk and angiotensin-converting enzyme inhibitors (ACEIs) should be saved for patients with diabetes or glomerulopathy [85]. Pazopanib is a TKI developed for VEGF 2 inhibition, but has multiple targets: VEGF 1 and 3, KIT, and PDGFR, involved in angiogenesis. It is indicated in RCC and soft tissue sarcoma with a safety profile that includes hypertension, proteinuria, thrombosis, hepatotoxicity, hypothyroidism, cardiac dysfunction, and hair and skin color changes as specific adverse reactions [53].

### 4.5. Cardiac Failure (CF)

Cardiac failure (CF) in TKI treatment is one of the most serious cardiac adverse reactions reported (Table 4). Cardiovascular events account for approximately 6.1% of TKI-related DDIs. This cardiotoxicity arises from both on-target and off-target effects of TKIs on cardiac tissue. One key mechanism involves the inhibition of kinases that are critical for cardiomyocyte survival and function. For example, TKIs targeting VEGFR can disrupt angiogenesis, leading to endothelial dysfunction and impaired myocardial perfusion. A reduced blood supply may contribute to ischemia and subsequent cardiac injury [86]. Moreover, TKIs may interfere with signaling pathways responsible for mitochondrial integrity and energy metabolism. Mitochondrial dysfunction leads to an accumulation of reactive oxygen species, inducing oxidative stress and triggering apoptotic cell death in cardiomyocytes [87]. Additionally, some TKIs can disturb ion channel regulation, resulting in arrhythmia and further compromising cardiac contractility. Furthermore, the disruption of cellular autophagy and the induction of myocardial fibrosis by TKIs may impair the heart’s ability to repair itself, promoting structural remodeling and functional decline over time. These multifactorial processes collectively reduce the cardiac output and contribute to heart failure. Understanding these mechanisms is essential for developing strategies to mitigate cardiotoxicity in patients undergoing TKI therapy [88]. Patras de Campaigno et al. evaluated the CF risk by searching VigiBase^®^, a WHO safety report database, and selected 15 TKIs which had at least 100 ICSRs (individual case safety reports). Dasatinib, imatinib, bosutinib, sunitinib, and nilotinib were found to have a higher risk of CF than other TKIs, probably because of the additional inhibition of ABL-1/2, a non-receptor tyrosine kinase with a role in cardiac failure and hypermetropy [89]. For example, co-administration of sunitinib with CYP3A4 inhibitors like ketoconazole can elevate drug levels, heightening the risk of heart failure. Lapatinib, when combined with QT-prolonging agents, raises the likelihood of arrhythmias and cardiac decompensation. Ponatinib may cause or worsen hypertension, especially when used with drugs like NSAIDs or corticosteroids, potentially leading to heart failure. Similarly, dasatinib, which can cause fluid retention, may induce congestive heart failure when used with agents like thiazolidinediones or steroids. These interactions underscore the importance of cardiovascular monitoring and medication review in patients on TKI therapy [89]. QTc prolongation occurs rarely but the probability increases if drugs that prolong QTc, like amiodarone, ciprofloxacin, sertraline, or quetiapine, are associated with TKIs that prolong QTc (crizotinib, gefitinib, lapatinib, nilotinib, pazopanib, sorafenib, sunitinib, vemurafenib) and CYP3A4 inhibitors. If clinically mandatory, an ECG should be performed 24 h before and 7 days after initiating TKI treatment [90].

### 4.6. Hepatotoxicity

Hepatotoxicity is a common adverse reaction in patients who undergo TKI treatment, and hepatic functions are monitored by dosing ASAT, ALAT, and total bilirubin (Table 4). TKIs can induce hepatotoxicity through several interrelated mechanisms. They may cause direct liver cell injury by impairing the mitochondrial function and increasing oxidative stress, leading to hepatocyte apoptosis [91]. Inhibition of specific kinases involved in cellular metabolism can disrupt bile acid transport, resulting in cholestasis and further liver damage [92]. Additionally, immune-mediated reactions may contribute to hepatotoxicity, where drug-induced modifications of liver proteins trigger inflammatory responses [93]. Metabolic activation to reactive intermediates can also initiate liver injury. Before deciding on a dose reduction, the clinician should consider if the increased values of ALAT (>120 IU/L) and/or bilirubin (>2.4 mg/dL (40 µmol/L)) are caused by liver metastasis or concurrent administration of drugs, like acetaminophen, that may enhance liver toxicity [94]. For instance, imatinib can interact with paracetamol, leading to an accumulation of toxic metabolites such as NAPQI and resulting in acute liver injury. Another example is erlotinib, which is primarily metabolized by the CYP3A4 enzyme. When combined with strong CYP3A4 inhibitors like ketoconazole or ritonavir, erlotinib levels increase significantly, raising the risk of liver toxicity, including elevated transaminases and, in rare cases, cholestatic hepatitis. Conversely, drugs like rifampin, a potent CYP3A4 inducer, can alter the metabolism of lapatinib (used in HER2-positive breast cancer), leading to the formation of potentially hepatotoxic metabolites or decreased hepatic clearance. These interactions may result in serious liver damage, including liver failure. Therefore, close monitoring of the liver function and an awareness of potential drug interactions are critical during TKI therapy [94].

**Table 4:** Management strategies and monitoring parameters for common tyrosine kinase inhibitor (TKI)-related adverse events.

Adverse Reaction	Monitoring Parameters	Management	Ref.
Nausea and/or vomiting	Ca^2+^, Mg^2+^, K^+^ levels Signs of dehydration	Take with food if drug PK allows Antiemetic treatment (5 HT3 antagonists, metoclopramide, ginger); pay attention to QT prolongation or 5HT_3_ antagonists	Rimassa et al. [95]
Stomatitis/mucositis		Good oral hygiene, non-alcoholic mouthwashes, consumption of non-irritating food; initiate antimicrobial/antifungal therapy if needed	Shyam Sunder et al. [96]
Diarrhea	Number of stools Ca^2+^, Mg^2+^, K^+^ levels Stool test for *C. difficile* Evaluate patient’s baseline bowel patten to assess the severity	Loperamide, probiotics, diosmectite, rehydration salts if needed, low fiber, low-fat diet, 8–10 glasses of water/day	Yang, J. C. H. et al. [97] Zhou et al. [98]
Constipation	Evaluate patient’s baseline bowel patten to assess the severity	High-fiber meals, lactulose (pay attention to fluid intake to avoid obstruction), irritant laxative only when needed. Pay attention to opioid association	Zhou et al. [73]
Peripheral edemas	Serum albumin	Compression socks, limb elevation, diuretics (DDI potential, electrolyte perturbances)	Girard et al. [57]
Hepatotoxicity	ASAT, ALAT, total bilirubin	Lower doses or treatment interruption, depending on grade	Qian et al. [99]
Body weight increase/decrease	Body weight	Dietary regime, physical activity; evaluate cachexia!	Kodama et al. [100]
Rash	First week—presents as sensory disturbances, erythema, and edema, followed by papulopustular eruptions in the second week and crusting in the fourth week	Hydrating creams applied 2 times/day; topical corticoid applications; antibiotic treatment if needed (tetracycline/minocycline); do not use alcoholic-based solutions; wash with lukewarm water, not hot water	Vogel et al. [101]
Hyperlipidemia	Total lipid panel, triglycerides, cholesterol at baseline	Dietary measures (low-fat meals, fish, grains, fruits, vegetables), statins (rosuvastatin, pravastatin preferred because of CYP3A4 metabolism of most of the TKIs)	Blais et al. [102]
Hypertension	Blood pressure	Angiotensin-converting enzyme inhibitors, angiotensin II receptor blockers or dihydropyridine calcium channel blockers; avoid non-dihydropyridine calcium channel blockers	Zhu et al. [103]
Ocular disorders: blepharitis, conjunctivitis, epiphora, periorbital rash	Ocular irritation, crusts on eyelids, excessive tear production	Warm compress, eyelid hygiene, corticosteroids, or anti-inflammatory medications	Agustoni et al. [104]
Hypothyroidism	TSH, FT4; assess baseline thyroid function	levothyroxine	Gabora et al. [105]
Cardiac failure	FEVS	Angiotensin-converting enzyme inhibitors, loop diuretics, potassium-sparing diuretics, beta blockers	AlShatnawi et al. [106]
QTc prolongation	EKG	Lower doses or interrupt/discontinue TKI treatment	Abu Rmilah et al. [107]
Fatigue	Brief Fatigue Inventory (BFI) questionnaire Functional Assessment of Cancer Therapy-Fatigue (FACT-F) Patient-Reported Outcomes Measurement Information System Cancer Fatigue Short Form (PROMISE-CF-SF)	Treat underlying causes: hypothyroidism, anemia, anxiety, depression Dexamethasone, methylphenidate, L-carnitine Drug holiday Nutrition and exercise therapy	Takahashi [108]

## 5. Emerging Strategies and Further Steps

As new TKIs appear and new indications are approved, the number of patients who will be treated increases, and clinicians should have tools that help them predict DDIs and offer ways to manage these interactions. Practical approaches would be DDI screening before prescription, real-time severity alerts, prediction of TKI-specific toxicities, adaptive dosing, and genotypic personalization. Alrowais et al. developed a sparrow search optimization with deep learning-based DDI prediction (SSODL-DDIP) technique for healthcare decision-making in big data environments, which considers various relationship and drug properties when predicting the potential for and severity of DDIs [109]. Thyroid dysfunction induced by sunitinib and sorafenib was predicted using a validated machine learning model [110], which could be a starting point for predicting other TKI adverse reactions and adjusting the dose from the beginning. Predicting the toxicity of anticancer therapy should also be a standard of care, as DPD testing is now for 5 FU treatment. Recommendations are that during the clinical studies for new drugs, genome-wide association studies (GWASs) should be performed to identify markers or risk scores that could predict a higher toxicity for specific patients [111].

Therapeutic drug monitoring of TKIs is advised for axitinib, dasatinib, imatinib, sunitinib, and pazopanib, with clear target concentrations for efficacy, and is under investigation, with promising results, for most of the other TKIs, as van der Kleij et al. mention. The exposure–response and exposure–toxicity relationships are relevant when assessing the need for TDM, to identify the patients at risk of severe toxicities [112]. Another study supplementarily recommends TDM for gefitinib, erlotinib, osimertinib, trametinib, vemurafenib, nilotinib, crizotinib, and afatinib [113]. Although being routinely used for antibiotics, immunosuppressants, or antiepileptic drugs, TDM is not often used in TKIs. The main barriers are a lack of experience/awareness with/of TDM among healthcare professionals and patients, the processing time for measurement and interpretation of the TDM result (because there are insufficient laboratories serving this function), and the financial costs [114].

## 6. Conclusions

Tyrosine kinase inhibitors (TKIs) are a cornerstone in the treatment of various cancers, yet their complex pharmacokinetics and potential for significant drug–drug interactions (DDIs) necessitate careful management. These interactions can alter drug efficacy, leading to subtherapeutic responses or heightened toxicity, thereby affecting patient outcomes. Given the narrow therapeutic index of many TKIs, interactions with cytochrome P450 enzymes, transport proteins, and acid-reducing agents must be meticulously monitored. Since many cancer patients receive multiple medications, clinicians must carefully evaluate potential DDIs to optimize treatment outcomes. Moreover, these interactions can be identified by clinicians using DDI checkers, including by clinical pharmacists who can inform the prescribing physician, or may be flagged by computerized alert systems integrated into clinical decision support software.

In conclusion, managing DDIs, performing therapeutic drug monitoring (TDM), and ensuring patient safety with TKI treatment require a comprehensive and multidisciplinary approach. To ensure patient safety, this should involve oncologists, clinical pharmacists, and other healthcare professionals. Collaboration among these experts facilitates the early identification of potential DDIs, individualized dosing strategies through TDM, and proactive management of side effects. Moreover, educating patients about potential interactions with food, over-the-counter medications, and herbal supplements further enhances safety. By integrating these strategies, healthcare teams can optimize treatment outcomes, minimize toxicity, and improve the overall safety and efficacy of TKI therapy.

## Figures and Tables

**Table 1:** Tyrosine kinase inhibitors (TKIs) approved by EMA (as of January 2025), main molecular targets, and approved clinical indications.

Indication	TKI	Molecular Target
Non-small-cell lung cancer (NSCLC)	Gefitinib	EGFR
	Erlotinib	EGFR
	Afatinib	EGFR, Her2, Her 4
	Osimertinib	EGFR (including T790M mutation)
	Dacomitinib	EGFR
	Alectinib	ALK
	Brigatinib	ALK
	Ceritinib	ALK
	Lorlatinib	ALK, ROS-1
	Crizotinib	ALK, ROS-1
	Entrectinib	ALK, ROS-1, TRK
Renal cell carcinoma (RCC)	Sunitinib	VEGFR, PDGFR, KIT
	Pazopanib	VEGFR, PDGFR, KIT
	Cabozantinib	MET, VEGFR, RET
	Axitinib	VEGFR
Hepatocellular carcinoma (HCC)	Sorafenib	VEGFR, PDGFR, RAF
	Regorafenib	VEGFR, PDGFR, KIT
	Cabozantinib	MET, VEGFR, RET
Breast cancer	Lapatinib	EGFR, Her 2
	Neratinib	EGFR, Her 2
	Abemaciclib	CDK4/6
	Palbociclib	CDK4/6
	Ribociclib	CDK4/6
Gastrointestinal stromal tumors (GISTs)	Imatinib	KIT, PDGFR
	Sunitinib	KIT, PDGFR, VEGFR
	Regorafenib	KIT, PDGFR, VEGFR
	Ripretinib	KIT, PDGFR
Thyroid cancer	Vandetanib	RET, VEGFR, EGFR
	Cabozantinib	MET, VEGFR, RET
	Lenvatinib	EGFT, VEGFR
Colorectal cancer	Regorafenib	KIT, PDGFR, VEGFR
Melanoma	Vemurafenib	BRAF V600E
	Dabrafenib	BRAF V600E
	Trametinib	MEK

EGFR—Epidermal Growth Factor Receptor, HER2—Human Epidermal Growth Factor Receptor 2, HER4—Human Epidermal Growth Factor Receptor 4, ALK—Anaplastic Lymphoma Kinase, ROS-1—c-ros Oncogene 1, TRK—Tropomyosin Receptor Kinase, VEGFR—Vascular Endothelial Growth Factor Receptor, PDGFR—Platelet-Derived Growth Factor Receptor, KIT—Stem Cell Factor Receptor, MET—Hepatocyte Growth Factor Receptor, RET—RET Proto-Oncogene, RAF—Rapidly Accelerated Fibrosarcoma Kinase, CDK4/6—Cyclin-Dependent Kinases 4 and 6, BRAF V600E—B-Raf Proto-Oncogene with V600E Mutation, MEK—Mitogen-Activated Protein Kinase Kinase.

**Table 2:** Clinical impact of PPIs or acid reducing agents (ARAs) on selected TKIs.

TKI	Interacting Drug	Impact of the DDI	Severity
Erlotinib	Omeprazole (and other proton pump inhibitors)	Increased gastric pH reduces drug solubility, leading to decreased absorption and lower plasma levels.	Major
Gefitinib	Antacids (e.g., aluminum hydroxide/magnesium)	Elevated gastric pH from antacids decreases solubility, resulting in reduced absorption.	Moderate
Pazopanib	Esomeprazole (and similar agents)	Concomitant PPI use significantly lowers pazopanib bioavailability due to pH-dependent solubility.	Major
Neratinib	Lansoprazole	Decreased absorption due to altered gastric pH, reducing bioavailability.	Major

DDI—drug–drug interaction, TKI—tyrosine kinase inhibitor.

**Table 3:** Clinically relevant drug–drug interactions (DDIs) involving tyrosine kinase inhibitors (TKIs) and suggested management strategies.

TKI Target	TKI	DDI	DDI Management
ALK	lorlatinib	+ strong CYP3A4 inhibitors: ↑ AUC and c_max_	Avoid combination; if not possible, ↓ lorlatinib dose to 75 mg
	crizotinib	+ strong CYP3A4 inhibitors: ↑ AUC; + strong CYP3A4 inducers: ↓ AUC	Avoid combination; if not possible, ↓ dose by 50% when co-administration with strong inhibitors is mandatory
	ceritinib	strong CYP3A4 inhibitor and weak CYP2C9 inhibitor → affects CYP3A4 substrates	Caution at co-administration with narrow-therapeutic-index drugs; monitor INR if co-administered with warfarin
EGFR	osimertinib	+ strong CYP3A4 inducers: ↓ AUC	Avoid combination; if not possible, ↑ dose to 160 mg
	erlotinib	+ strong CYP3A4 inhibitors/inducers, P-gp inhibitors, coumarin-derived anticoagulants, cigarette smoke	Initial dose of 300 mg for current smokers instead of 150 mg for non-smokers; ↑ dose by 50 mg if association with strong inducers is mandatory; ↓ dose by 50 mg if association with strong inhibitors is mandatory
VEGFR	sorafenib	+ strong CYP3A4 inducers	Avoid combination; if not possible, dose adjustment
	regorafenib	+ strong CYP3A4 inducers, inhibitors	Avoid combination; if not possible, dose adjustment
	cabozantinib	+ strong CYP3A4 inducers, inhibitors	Avoid combination; if not possible, ↑ dose by 20 mg if association with strong inducers is mandatory; ↓ dose by 20 mg if association with strong inhibitors is mandatory
	apatinib	+ strong CYP3A4 inducers	Avoid combination; if not possible, dose adjustment
Multiple targets	imatinib	+ strong CYP3A4 inducers	Avoid combination; if not possible, ↑ dose by 50% if association with strong inducers is mandatory
	pazopanib	+ strong CYP3A4 inhibitors/inducers, P-gp or BCRP inhibitors	Avoid combination; if not possible, ↓ dose by 50% when co-administration with strong inhibitors is mandatory

+ AUC—area under the curve, strong CYP3A4 inhibitors (itraconazole, ketoconazole), strong CYP3A4 inducers (rifampicin), ↑ increase, ↓ decrease.

## Data Availability

No new data were created or analyzed in this study. Data sharing is not applicable to this article.

## Abbreviations

The following abbreviations are used in this manuscript:

VEGF	vascular endothelial growth factor
TKI	tyrosine-kinase inhibitor
CML	chronic myeloid leukemia
DDIs	drug–drug interaction
CYP450	cytochrome P450
P-gp	P-glycoprotein
RTKs	tyrosine-kinase receptors
EGFRs	epidermal growth factor receptors
VEGFRs	vascular endothelial growth factor receptors
PDGFRs	platelet-derived growth factor receptors
ROR1, ROR2	receptor tyrosine kinase-like orphan receptors 1 and 2
NSCLC	non-small-cell lung cancer
RCC	renal cell carcinoma
HCC	hepatocellular carcinoma
GIST	gastrointestinal stromal tumor
PPIs	proton pump inhibitors
OS	overall survival
PFS	progression-free survival
mRCC	metastatic renal cell carcinoma
ABC	ATP-binding cassette
BCRP	breast cancer resistance protein
CNS	central nervous system
c_max_	maximum plasma concentration
PXR	pregnane X receptor
OATP1B1	organic anion transporting polypeptide 1B1
ALK	anaplastic lymphoma kinase
INR	international normalized ratio
CPK	creatine-phosphokinase
CAM	complementary and alternative medicine
CTCAE	Common Terminology Criteria for Adverse Events
AE	adverse event
ALAT	alanine aminotransferase
ASAT	aspartate aminotransferase
EKG	electrocardiogram
MET	mesenchymal epithelial transition factor receptor
CF	cardiac failure
ICSR	individual case safety report
TDM	therapeutic drug monitoring
